# Important Role of CYP2J2 in Protein Kinase Inhibitor Degradation: A Possible Role in Intratumor Drug Disposition and Resistance

**DOI:** 10.1371/journal.pone.0095532

**Published:** 2014-05-12

**Authors:** Céline Narjoz, Amélie Favre, Justin McMullen, Philippe Kiehl, Michael Montemurro, William D. Figg, Philippe Beaune, Isabelle de Waziers, Bertrand Rochat

**Affiliations:** 1 Université Paris Descartes, INSERM UMR S-U775, Sorbonne Paris Cité, Paris, France; 2 Hôpital Européen Georges Pompidou, Service de Biochimie, Unité Fonctionnelle de Pharmacogénétique et Oncologie Moléculaire, Paris, France; 3 Quantitative Mass Spectrometry Facility, Centre Hospitalier Universitaire Vaudois, Lausanne, Switzerland; 4 University Hospital Zürich, Oncology, Zürich, Switzerland; 5 Genitourinary Malignancies Branch, Center for Cancer Research, National Cancer Institute, National Institutes of Health, Bethesda, Maryland, United States of America; University of Nebraska Medical Center, United States of America

## Abstract

We have investigated *in vitro* the metabolic capability of 3 extrahepatic cytochromes P-450, CYP1A1, 1B1 and 2J2, known to be over-expressed in various tumors, to biotransform 5 tyrosine kinase inhibitors (TKI): dasatinib, imatinib, nilotinib, sorafenib and sunitinib. Moreover, mRNA expression of CYP1A1, 1B1, 2J2 and 3A4 in 6 hepatocellular and 14 renal cell carcinoma tumor tissues and their surrounding healthy tissues, was determined.

Our results show that CYP1A1, 1B1 and especially 2J2 can rapidly biotransform the studied TKIs with a metabolic efficiency similar to that of CYP3A4. The mRNA expression of CYP1A1, 1B1, 2J2 and 3A4 in tumor biopsies has shown i) the strong variability of CYP expression and ii) distinct outliers showing high expression levels (esp. CYP2J2) that are compatible with high intratumoral CYP activity and tumor-specific TKI degradation.

CYP2J2 inhibition could be a novel clinical strategy to specifically increase the intratumoral rather than plasma TKI levels, improving TKI efficacy and extending the duration before relapse. Such an approach would be akin to beta-lactamase inhibition, a classical strategy to avoid antibiotic degradation and resistance.

## Introduction

Both *in vitro* and *in vivo* data have highlighted numerous systemic or intratumoral mechanisms of resistance to anticancer agents like the targeted tyrosine kinase inhibitors (TKI) [1]. One of them is the degradation of TKI by xenobiotic (drug) metabolizing enzymes (XME) [2]. Studies on the fate of TKI in humans indicate that they are rapidly biotransformed to inactive compounds by various hepatic XME, mainly cytochrome P-450 isozymes (CYP) [3]. But the importance of CYP-mediated *intratumoral* degradation of TKI with consequences in terms of treatment efficacy, and resistance is largely unknown whereas tumor-expressed metabolizing enzymes (TEME) can increase cancer cell resistance by significantly lowering intratumoral drug concentration [4].

Interestingly, the XME expression pattern found in the liver [5], the principal organ involved in TKI systemic degradation, is not similar to the pattern found in tumors [6], [7], [8]. For instance, CYP3A4 is the major XME involved in metabolism of drugs including TKI [3], in terms of moles of enzymes, number of drug-substrates and metabolism velocity in the liver. But CYP3A4 is usually very poorly expressed in tumor biopsies and cell lines [9]. By contrast, CYP1B1, 2J2 and 1A1 are extra-hepatic enzymes because of their weak mRNA and protein expression in the liver [5] whereas they can be highly expressed in various cancer cell lines or resected tumors [6], [7], [10], [11], [12], [13].

Together with influx (solute carrier, SLC) and efflux (ATP-binding cassette pumps, ABC) membrane systems and through a synergistic interplay, TEME are involved in i) the decrease of intratumoral/blood drug level ratio via the increase of intratumoral drug clearance and ii) the appearance of drug resistance [4]. These defense mechanisms have been built over millions years of vertebrate evolution with ABC and XME systems as the corner stone of cell detoxification [14], [15].

Interestingly drug-dependent resistance mechanisms can be transient allowing some cancer cell sub-clones to survive with stabilized growth (dormancy [16]) until new genetic modifications allow escaping the drug efficacy via drug- and target-independent mechanism(s) of resistance [4]. It underlines the interest to study the intratumoral disposition of TKI.

In this study, we have evaluated the capability of CYP1A1, 1B1 and 2J2, known to be over-expressed in various tumor types [6], [7], [10], [11], [12], [13] and known to biotransform various drugs [17], [18], [19], [20], to metabolize 5 TKI: dasatinib, imatinib, nilotinib, sunitinib and sorafenib. Determination of TKI and TKI metabolites levels were determined by liquid-chromatography mass spectrometry (LC-MS). TKI disappearance in i) *in vitro* microsomal incubations containing cDNA expressed CYP isozymes and ii) culture media from HepG2 cells infected with adenovirus expressing CYP mRNA and proteins, have been performed. Michaelis-Menten constants, Km, Vmax and intrinsic clearance (IC), were calculated. The catalytic activity of CYP1A1, 1B1 and 2J2 on TKI biotransformation was compared to the one of CYP3A4, which is the main hepatic isozyme involved in TKI biotransformation [3].

Finally, mRNA expression of CYP1A1, 1B1, 2J2 and 3A4 was established in 6 hepatocellular carcinoma (HCC) and 14 renal cell carcinoma (RCC) tumor biopsies and their healthy tissue counterparts surrounding the tumor. This allowed for the evaluation of the potential real impact that these TEME could play on TKI intratumoral levels.

## Results

### TKI degradation in *in vitro* microsomal incubations

**Figure 1:**
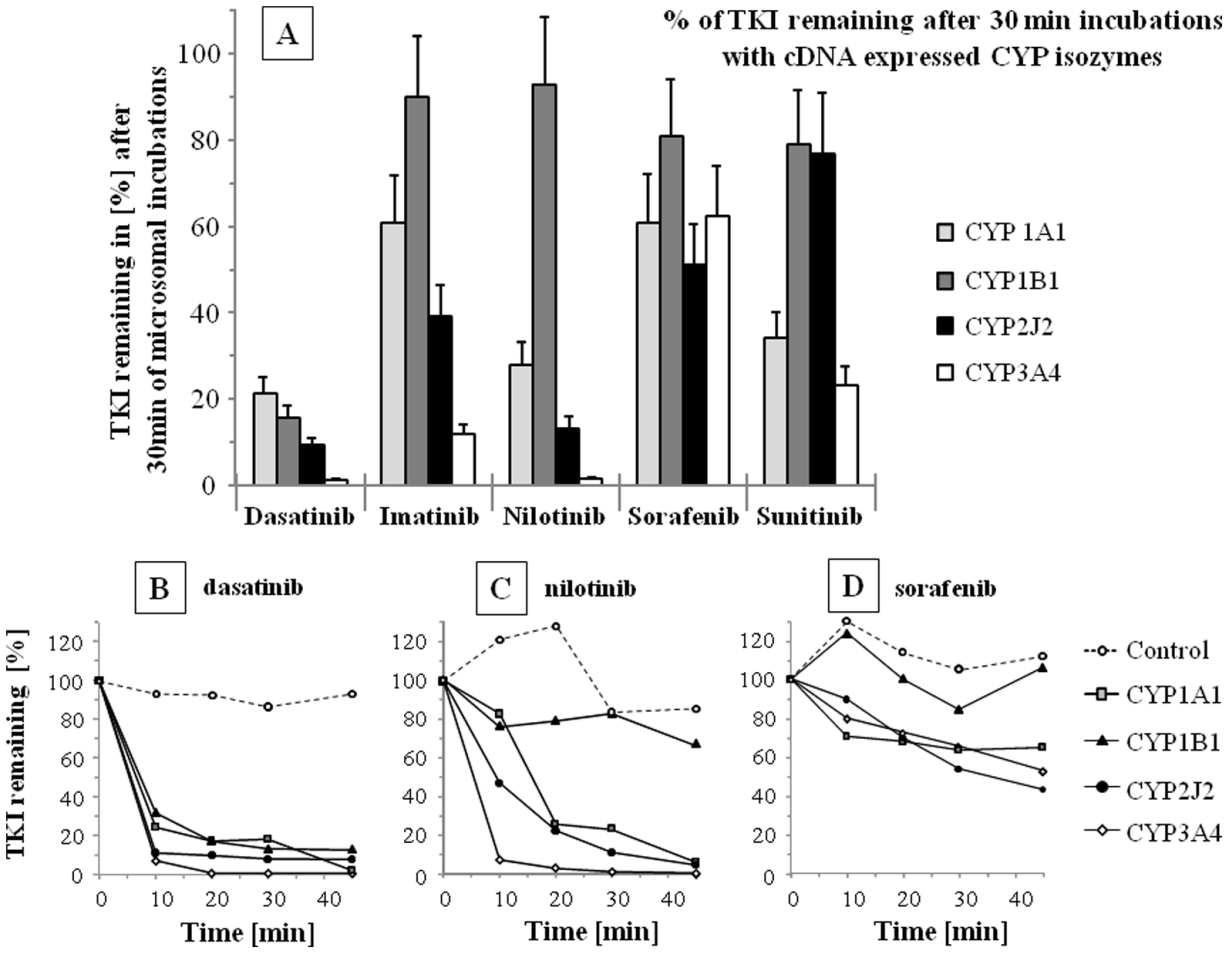
Disappearance of TKI in *in vitro* incubations. Disappearance of dasatinib, imatinib, nilotinib, sorafenib and *Z*-sunitinib (each TKI = 2.5 µM at T = 0) from *in vitro* incubations performed with cDNA expressed CYP1A1, 1B1, 2J2 and 3A4 isozymes. A) Remaining TKI in the microsomal incubations after 30 min., are given in percent of initial TKI levels (T = 0; SD corresponds to the maximum analytical deviation found in the LC-MS assays). Time course of B) dasatinib, C) nilotinib and D) sorafenib degradations in the incubations are depicted for cytochrome P-450 (CYP) 1A1, 1B1, 2J2 and 3A4 isozymes.

**Figure 1A** depicts the percentage of TKI remaining in microsomal incubations performed with cDNA expressed CYP after 30 min. In control incubations, TKI amounts did not decrease significantly (85–115% of TKI amount at T = 0). The full time courses of dasatinib, nilotinib and sorafenib are presented in **Figure 1B, C and D** respectively. CYP2J2 and 3A4 activities have the highest degradation rate for most TKI (**Figure 1A**). After 30 min, more than 60% of dasatinib, nilotinib and imatinib levels were removed from incubations. The disappearance rate was calculated after 10 min (see **Table 1**) taking into account the percent of TKI removed from the incubation (0.25 nmole of TKI and 20 pmoles of CYP in 100 µL) and are expressed in (nmole of degraded TKI/(nanomole of CYP×min)). These results show that TKI degradation mediated by CYP1A1, 1B1 or 2J2 activity is in the same order of magnitude than CYP3A4 (**Table 1**).

**Table 1:** Disappearance rates of TKI.

	Disappearance rate in microsomal incubations				
	nmoles of TKI degraded/(min×nmole of CYP)				
CYP	Dasatinib	Imatinib	Nilotinib	Sorafenib	Sunitinib
**1A1**	0.9	0.3	0.2	0.4	0.6
**1B1**	1.0	0.2	0.3	0.0	0.1
**2J2**	1.1	0.3	0.7	0.1	0.3
**3A4**	1.2	0.8	1.2	0.2	0.6

Disappearance rates of TKI (2.5 µM at T = 0) in microsomal incubations in the first 10 minutes. Microsomal incubations were performed with 0.25 nM of TKI, 20 picomoles of CYP1A1, 1B1, 2J2 or 3A4. The disappearance rate is expressed in nmole of TKI degraded per min and nmole of CYP.

### *In vitro* enzyme kinetic study

First, TKI metabolites were identified (see *Material and Method*). For instance, the formation of 2 nilotinib oxidized metabolites, mass-to-charge ratio (m/z) at 546.3, were revealed by LC-MS analysis. Five MS^2^ product ions were similar in the fragmentation of nilotinib and these 2 metabolites (**Figure S1**). Using GraphPad Prism software, the affinity (Km) and maximum velocity (Vmax) of metabolite production were calculated (**Figure S1** for nilotinib metabolites against nilotinib concentrations for CYP2J2 and 3A4).

**Table 2** shows the calculated Km values of CYP1A1, 1B1, 2J2 and 3A4 for the identified metabolites. In **Table 2**, we can observe that, in contrast to CYP1A1, 1B1 and 2J2, CYP3A4 is capable of producing *all* identified metabolites, indicating low substrate specificity of CYP3A4 and high chemical variability of metabolites produced by this isozyme. CYP1A1 and 2J2 did produce most of the studied metabolites even if, in some cases, the intrinsic clearance values were lower than the CYP3A4 intrinsic clearance (relative IC<1). In some cases, CYP1A1, 1B1 and 2J2 produced some TKI metabolites with a much higher IC than CYP3A4. For instance, CYP1A1 showed a relative IC of 2.6, 2.9 and 28 times higher for OH-imatinib #3, #5 and NO-imatinib #7, respectively (**Table 2**). IC for OH-imatinib #5 controlled by CYP1B1 was 4.5× higher than mediated by CYP3A4 (**Table 2**). Finally, CYP2J2 showed an equal, 0.9×, or higher IC, 4.0×, 1.3×, 3.6×, 3.9×, for, respectively, OH-imatinib #6, OH-imatinib #7, NO-nilotinib, OH-nilotinib and OH-sorafenib than CYP3A4.

**Table 2:** Michaelis-Menten kinetic parameters.

		Affinity Constant, Km [µM]				Relative Intrinsic Clearance [fold diff.]			
PKI	metabolites	CYP				CYP			
		1A1	1B1	2J2	3A4	1A1	1B1	2J2	3A4
**Dasatinib**	dehydroxyethyl-dasatinib	4.7	2.2	3.9	71.4	0.5	1.1	0.5	1.0
**Imatinib**	N-demethyl-imatinib	7.6 (*)	/	1.7	2.3	0.2 (*)		0.4	1.0
	OH-imatinib #3	3.4 (*)	/	/	3.4	2.6 (*)			1.0
	OH-imatinib #5	4.0 (*)	5.5 (*)	2.3	3.1	2.9 (*)	4.5 (*)	0.3	1.0
	NO-imatinib #6	/	/	0.6	2.2			0.9	1.0
	NO-imatinib #7	1.0 (*)	7.5 (*)	0.4	5.3	27.9 (*)	0.6 (*)	4.0	1.0
	NO-imatinib #8	/	/	/	2.9				1.0
**Nilotinib**	NO-nilotinib #1	3.0	/	0.8	27.6	0.5		1.3	1.0
	OH-nilotinib #2	4.0	/	0.7	9.8	0.0		3.6	1.0
**Sorafenib**	NO-sorafenib #1	/	/	/	6.1				1.0
	OH-sorafenib #2	1.6	/	1.5	5.4	0.2		3.9	1.0
**Sunitinib**	*E*-deethyl-sunitinib #1	6.9	2.7	89.1	13.8	0.1	0.1	0.2	1.0
	*Z*-deethyl-sunitinib #2	6.7	2.7	80.7	13.2	0.2	0.1	0.2	1.0

Michaelis-Menten kinetic parameters determined in microsome incubations with cDNA expressed CYP1A1, 1B1, 2J2 and 3A4 isozymes. A) Affinity constants, Km in µM, for different TKI metabolic pathways. B) Relative intrinsic clearance (Vmax/Km; in arbitrary unit [arb]) for the same TKI metabolic pathways, expressed as fold differences of the intrinsic clearance of CYP3A4, the major hepatic enzymes involved in TKI degradation. Abbreviations: OH-: hydroxyl-metabolites, NO-: N-oxide metabolite. Imatinib metabolites are numbered in accordance to [18].

(*) Results obtained in our previous published study [18].

### TKI degradation in media of CYP infected cells

**Figure 2:**
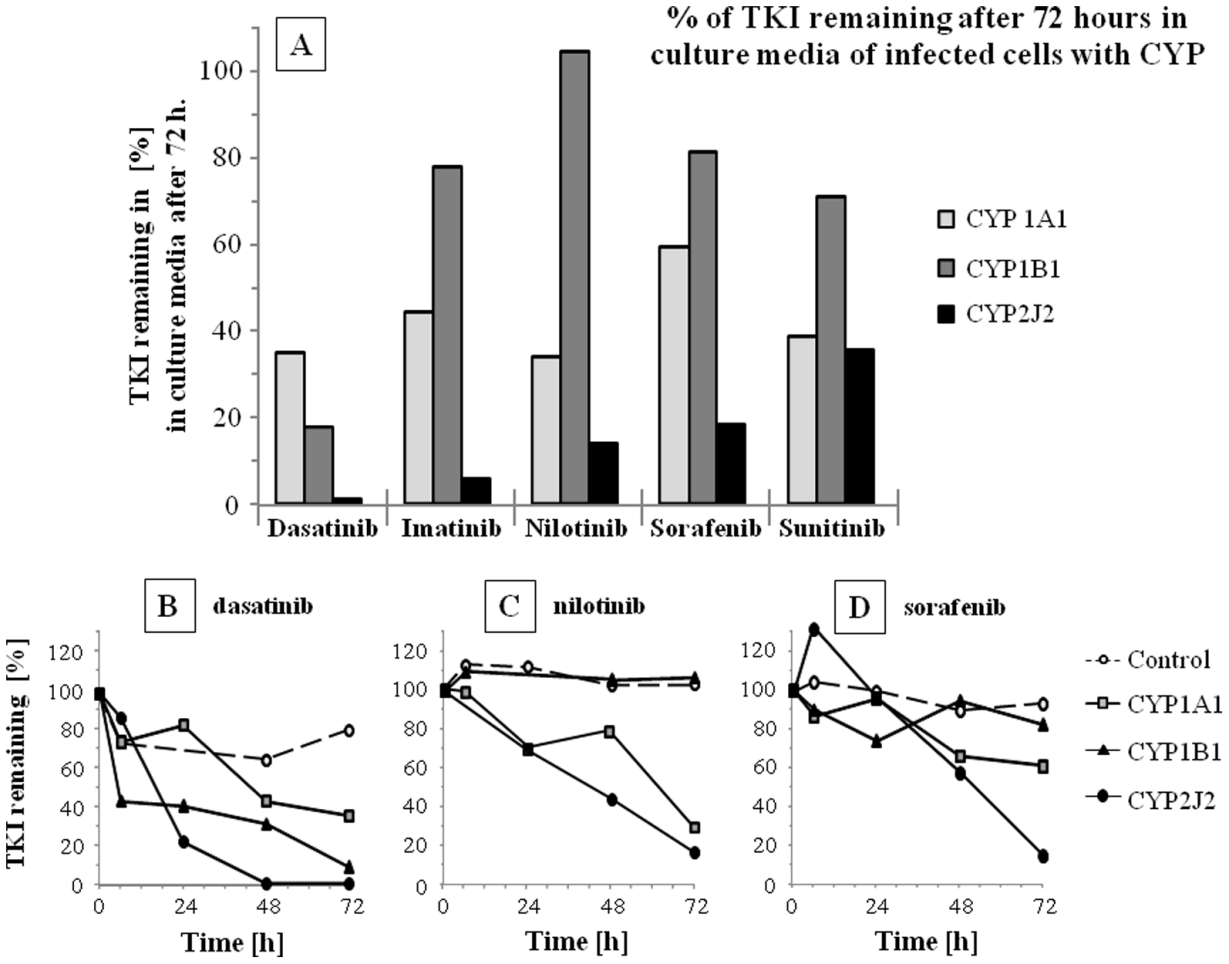
Disappearance of TKI in in culture media. Disappearance of TKI (2.5 µM added in the culture media at T = 0) in culture media of HepG2 cell infected with lacZ (control), CYP1A1, 1B1, 2J2 and 3A4. Disappearance of TKI was determined by LC-MS analysis and is given in % of the levels at T = 0. A) Remaining TKI in the culture media after 72 hours (representative experiment from 3 experiments in percent of initial TKI levels). Time course of B) dasatinib, C) nilotinib and D) sorafenib disappearance in the incubations are depicted for cytochrome P-450 (CYP) 1A1, 1B1 and 2J2 isozymes.

**Figure 3:**
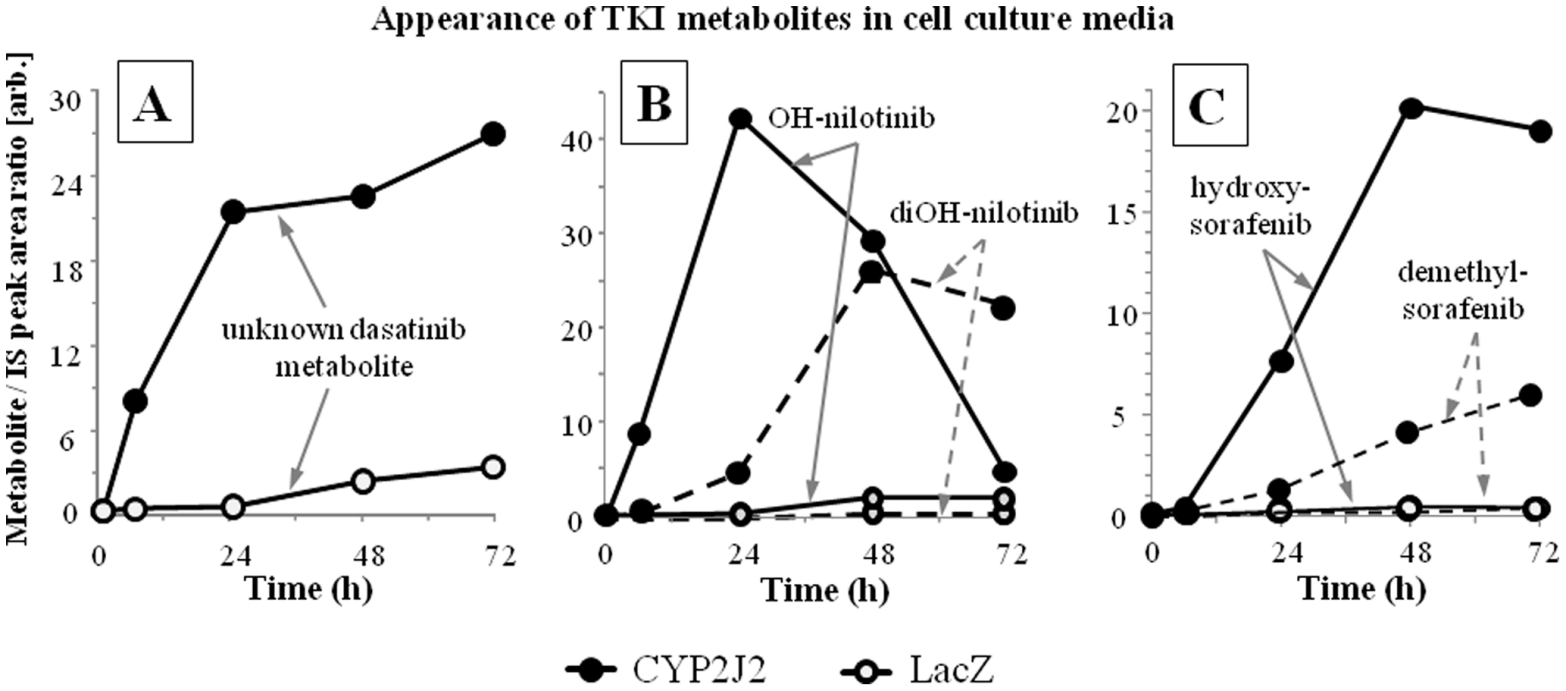
Appearance of TKI metabolites in culture media. Appearance of TKI metabolites in culture media from CYP2J2 and LacZ infected cells. Production of TKI metabolite is given in arbitrary unit corresponding to metabolite/IS LC-MS peak area ratio. A) Appearance of a dasatinib metabolite that was not formally identified with a pure standard. The m/z value indicates that it should be a hydroxy-desaturated dasatinib; B) Appearance of nilotinib hydroxy-metabolites of the first and second generations with one and two oxidations, respectively. C) Appearance of nilotinib hydroxy-sorafenib in cell culture media.

The TKI disappearance in the culture media was expressed as LC-MS peak area remaining in comparison to T = 0 (**Figure 2A**). The full time courses of dasatinib, nilotinib and sorafenib are presented in **Figure 2B, C and D** respectively. Whereas there was a relatively large variability in TKI disappearance between the 4 experiments, the data obtained confirms the results obtained with the microsomal incubations (**Figure 1A** **and** **Table 1**). It underscores the strong capacity of CYP2J2 to biotransform the 5 TKI tested. In HepG2 cell culture media, dasatinib is removed by all tumor-expressed CYP with the following clearance rate: CYP2J2>CYP1A1>CYP1B1. Imatinib, nilotinib and sorafenib are mainly removed by CYP2J2, to a lesser extent by CYP1A1 and very slowly by CYP1B1. Z-sunitinib is removed from the culture media at equal rate for CYP1A1 and 2J2. In most cases, the TKI disappearance could be linked to the appearance of 1^st^ and 2^nd^ generation metabolites in the culture media (**Figures 3A–C**).

### mRNA expression in tumor and surrounding healthy tissues

**Figure 4:**
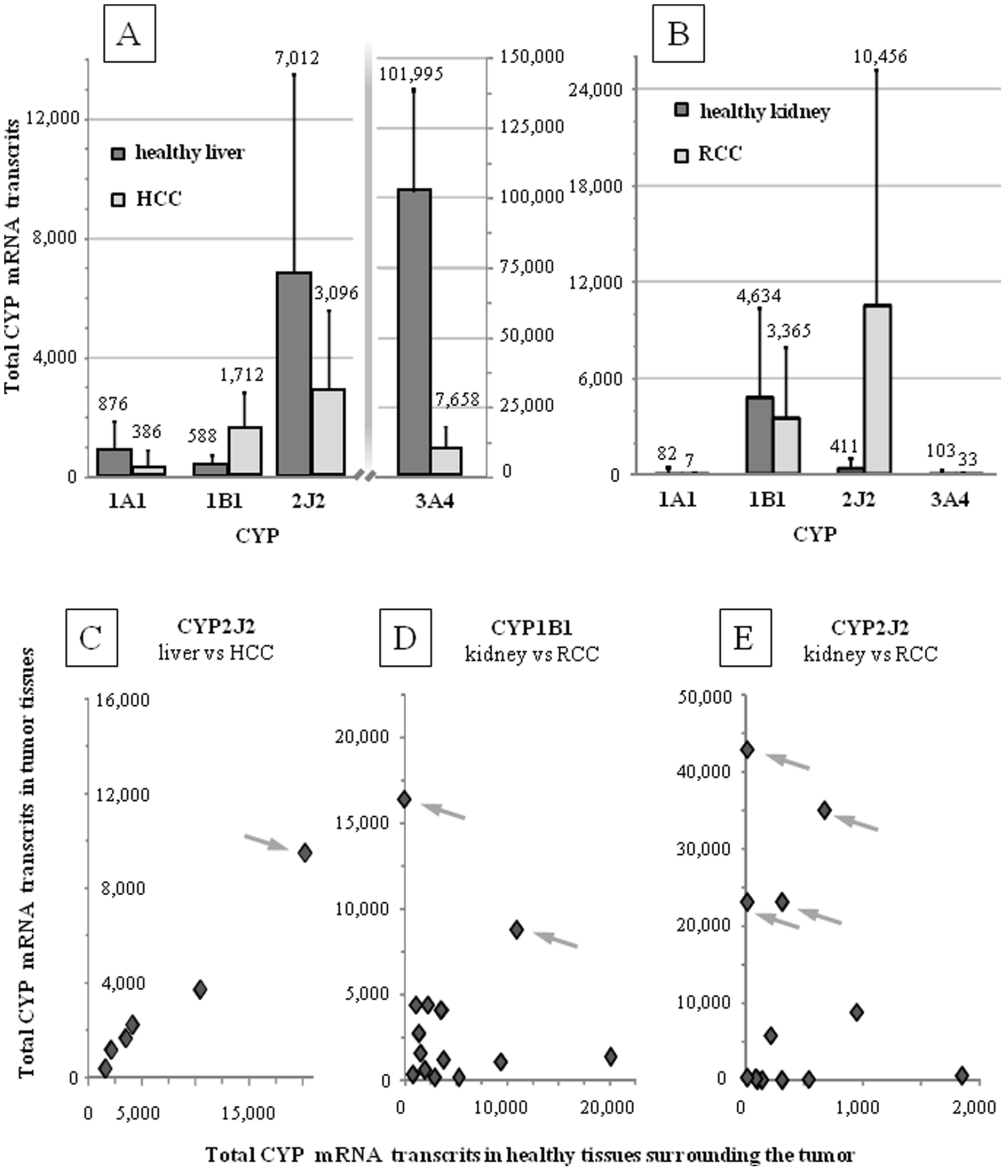
mRNA relative expression of CYP1A1, 1B1, 2J2 and 3A4. Mean +/− SD of the total mRNA relative expression of CYP1A1, 1B1, 2J2 and 3A4 in A) hepatocellular carcinoma (HCC; N = 6 and B) renal cell carcinoma (RCC; N = 14) and the healthy tissues counterpart surrounding the tumors. Correlation between total mRNA expression found in healthy tissues for C) CYP2J2 for HCC and for D) CYP1B1 for RCC and E) CYP2J2 for RCC. Outliers with very high mRNA expression can be observed and are highlighted with arrows.

Total CYP mRNA expression was studied in 6 HCC, 14 RCC and their surrounding healthy tissue counterparts (≥∼85% of cancer and ≥∼90% healthy cells, respectively). Mean values +/− SD are presented in **Figures 4A and 4B** (see all values in **Table S1**). Total CYP mRNA transcripts in tissues have been determined according to a methodology previously described [5], [21] that allows for the comparison of both results. The mean mRNA expression of CYP1B1 in HCC and CYP2J2 in RCC were significantly higher than in the surrounding healthy tissue counterparts (*p* values = 0.023 and 0.028, respectively).

Individual transcript number in tumor and surrounding healthy tissues are depicted in **Figures 4C to 4E** (see values in **Table S1**). One HCC tissue shows a unique and high level of CYP2J2 expression (**Figure 4C**). CYP1A1 and 1B1 transcripts in HCC tissues were very low and no clear outliers could be seen (****). **Figures 4D and 4E** show the mRNA expression of CYP1B1 and 2J2 in RCC tumor and healthy tissues. Interestingly, in 2 HCC and 4 RCC tissues, the number of transcripts was very high, reflecting a high inter-individual variability. In contrast, in half of the HCC and RCC tissues, CYP1B1 and 2J2 were poorly expressed. CYP1A1 and 3A4 mRNA expression was very low in RCC tissues.

## Discussion

By measuring TKI disappearance in microsomal incubations (**Figure 1A–D** and **Table 1**) or cell culture media (**Figure 2A–D**), and determining kinetic parameters (Km and IC, **Table 2**), we report that CYP1A1, 1B1 and, for the first time, CYP2J2 were able to degrade TKI with comparable efficiency than CYP3A4, the major isozyme involved in TKI systemic clearance. These clearance rates are comparable with previously published data on gefitinib, erlotinib and dasatinib [19], [20]. In addition, Kamath et al. [22] found that, in similar microsomal incubations, dasatinib was degraded by CYP1A1, 1B1 and 3A4 with comparable rate of disappearance (CYP2J2 was not tested). In addition, most Km values reveal a strong affinity of the TKI-CYP complexes (**Table 2**; values in the low micromolar range). The determined Km values of TKI for CYP3A4 were compared with available data (differences up to 3×) [15], [23], [24], [25], [26], [27]. Thus, our data confirm the excellent affinity of the studied TEME for dasatinib, imatinib, nilotinib, sorafenib and sunitinib.

The potential intratumoral impact of these 3 CYPs was revealed by determining the mRNA expression levels in tumor biopsies and their healthy tissue counterparts. Data obtained in this study and in Bièche et al. [5] are consistent and show that CYP3A4 has a very high number of transcripts in the liver but a very low number in the kidney (**Figure 4A and 4B**). As expected, CYP1A1, 1B1 and 2J2 have a medium or low expression in the liver (**Figure 4A**).

When mean values of the number of total transcripts are considered, there are only a few conclusions that can be established (**Figures 4A and 4B**). First, CYP1B1 and 2J2, were significantly over-expressed in the HCC and RCC, respectively in comparison to the healthy tissue. Secondly, CYP3A4 was significantly down-regulated in HCC tissues. Thirdly, the mean absolute values of total transcripts in tumor samples, is relatively low for CYP1A1, 1B1 in the HCC as well as for CYP1A1, 1B1 and 3A4 in the RCC.

The crucial information is revealed when individual values are considered (**Figures 4C–E**). First, except for CYP2J2 in HCC (**Figure 4C**), there are no correlations between CYP expression in tumor and healthy tissues (**Figure 4D–E**). This is consistent with previous reports showing that ABC and XME enzyme expressions are not or poorly associated with tumor type [28]. Secondly, there are many outliers that show very high number of CYP transcripts in tumor (see grey arrows in **Figures 4C**–**E**). The mRNA expression of these outliers' was comparable with the expression level of CYP implicated in systemic drug biotransformation and clearance (e.g. CYP3A4 in healthy liver). For instance, in one RCC tissue, the number of CYP2J2 transcripts was similar with the number of CYP3A4 transcripts in healthy hepatic tissues (**Figure 4E**, **Table S1** and patient #2529) [5].

These results underscore the versatility of CYP expression in tumors and the possibility of some cancer subclones to very rapidly induce CYP in order to detoxify the cancer cell from “TKI-toxins”, leading to survival advantage.

Both pre-existing and acquired resistant clones can lead to treatment relapse. Using cell culture as a study model, Scappini et al. [30] have shown that in one of the studied cell line, the resistance to imatinib occurred linearly and gradually indicating probable induction of various mechanisms rather than target mutations or sudden appearance and growth of a subclone pre-existing to drug treatment (observed in a second cell line). This underscore that acquired resistance, including intratumoral drug disposition, should also be considered for possible clinical interventions.

The fate of TKI anticancer agents has mainly been studied in relation to hepatic xenobiotic metabolizing enzymes (XME) and pharmacokinetics in plasma. Today, there is increasing data from literature showing the importance of XME-mediated intratumoral bioavailability [2], [4]. First, and in this context of intratumoral drug disposition, efflux transporters have been in the forefront of the scene. However, attempts to inhibit efflux transporters *in vivo* (P-gp, MDR-1 and ABCB1) in order to increase intracellular drug disposition have thus far been unsuccessful [29]. Because of its poor tumor specificity, inhibition of ABCB1 efflux transporter in humans has caused strong side effects, preventing this to be a viable treatment option.

In contrast, the CYP isozymes studied here, CYP1A1, 1B1 and 2J2, are poorly expressed in the liver (**Figures 4A** and [5]) and the consequences of their inhibition should not be systemic but rather located at their tissue of expression, which is low in comparison to the expression of CYP1A2, 2C8, 2C9 and 3A4 in the liver [5]. CYP1A1 and CYP1B1 mRNA transcripts have been mainly found in lung and trachea and in prostate and uterus, respectively. CYP2J2 mRNA transcripts have been found mainly in jejunum and ileum and, to a lesser extent, in liver, skeletal muscle, heart and colon [5].

*In vivo* modulation of tumo-expressed metabolizing enzyme (TEME) activity appears promising because, in contrast to the ubiquity of ABCB1 efflux pump, it should have limited side effects on other tissues/organs. Inhibition of TEME should increase intratumoral drug levels specifically and have similar consequences than dose escalation, one usual practice when drug resistance occurs in patients [31], but with no significant increase of TKI plasma levels. In *in vivo* models, modulation of anticancer agent disposition by modifying intratumoral CYP activity has been already done with success (gene-directed enzyme prodrug therapy). The anticancer agent levels were significantly increased intratumorally whereas plasma levels were identical between control and treated animals [32], [33].

It has been reported that the synergistic interplay between XME in rapid multistep drug metabolism and between XME and efflux systems, can reduce intracellular drug levels and efficacy by much more than 10 times [4]. It underscores the coordination and efficiency of detoxification mechanisms in cell and organism survival.

Moreover, in cancer cells, genomic alteration seems to be, in some cases, organized (e.g. transposable elements) [34] and not random. It can induce cell defence and survival mechanisms related to non-canonical and extreme over-expression (>100×) of XME or/and ABC systems. This has been reported for human CYP1A1 and ABCB1 [35], [36] as well as for ABC and XME systems in other living systems where resistance to xenobiotics appeared [37], [38].

In addition to the capacity of CYP1A1, 1B1 and 2J2 to biotransform various drugs including TKI, it has been reported that these 3 CYPs are able to biotransform arachidonic acid to second messengers that show anti-apoptotic effects (e.g. epoxyeicosatrienoic acids, EET) [39]. Thus, the reduction of anti-apoptotic compound synthesis could be an additional reason to give CYP1A1, 1B1 and/or 2J2 inhibitors.

In conclusion, the results presented here show that CYP1A1, 1B1 and especially CYP2J2 can rapidly metabolize dasatinib, imatinib, nilotinib, sorafenib and sunitinib with similar disappearance velocities than CYP3A4. The determination of mRNA expression of CYP1A1, 1B1, 2J2 and 3A4 in tumor biopsies have mainly shown the strong variability of CYP expression with distinct outliers showing high expression levels that are compatible with high intratumoral CYP activity. This strongly suggests that the inhibition of these tumor-expressed metabolizing enzymes (TEME) could increase mainly the intratumoral rather than plasma drug levels with, possibly, mild side effects. Indeed, CYP2J2 inhibitors have already been identified among approved drugs (e.g. telmisartan, flunarizine, danazol, and amiodarone [40], [41]) and their safety profile has been established in human. Inhibition of the tumor-expressed metabolizing enzyme CYP2J2, could be a novel clinical strategy to improve TKI efficacy and to decrease the delay before relapse reflecting beta-lactamase inhibition that is a classical strategy to avoid antibiotic degradation and resistance.

## Materials and Methods

### Chemicals

Chromatography was performed using HPLC grade solvents obtained from Sigma-Aldrich (Switzerland). All other chemicals were of analytical grade. Ultrapure water was obtained from a Milli-Q® UF-Plus apparatus (Millipore Corp., USA). Imatinib, nilotinib, dasatinib, sunitinib (Z-isomer) and sorafenib were kindly provided by Novartis Pharma AG (Switzerland), Bristol–Myers Squibb (Switzerland), Pfizer and Bayer. Stable-isotope internal standards (IS) were kindly supplied by Pharma companies or purchased from Sigma-Aldrich or Alsachim (France). Control, cDNA expressed CYP microsomes and incubation buffers were purchased from BD Bioscience (Belgium).

### *In vitro* microsomal incubation

TKI degradation (time course) and TKI metabolite production (determination of Km and Vmax constants) were performed by *in vitro* microsomal incubations prepared in 0.5 mL Eppendorf vials. Incubations with a final volume of 100 µL, were performed according to BD Gentest procedure/catalog (BD Bioscience, Belgium) and contained human cDNA insect cell microsomes expressed CYP isozymes and insect cell control microsomes without CYP activities (BD Bioscience, Belgium). Each incubation contained the regenerating solutions A and B, 0.1 M phosphate buffer at pH 7.4, 50 µg of proteins (control microsomes), 20 pmoles of CYP isozymes with TKI at final concentrations of 2.5 µM for degradation experiment and 10 pmoles of CYP isozymes with 0.5, 1, 2, 5, 10 and 20 µM for Km and Vmax constant determination. Incubations were prepared on ice, started when vials were placed in a 37°C water bath and stopped by addition of 2 volumes of MeCN at 4°C. For Km and Vmax constants determination, the incubations were stopped at 15 min. For the degradation time course, 10 µL incubation was withdrawn at 0, 10, 20, 30 and 45 min and transferred directly to 0.5 mL Eppendorf. With 10 pmoles of CYP, the production rate of metabolites was linear for at least 20 minutes.

### Determination of Km and Vmax constants

Michaelis-Menten constants were determined in the microsomal incubations as described above and previously [15]. A minimum of 2 experiments with at least 4 out of 6 TKI concentrations were taken into account for the calculation of Vmax and Km. The TKI concentrations (0.5 to 20 µM) correspond to the levels found in plasma or in peripheral blood mononuclear cells (surrogate of imatinib, nilotinib and dasatinib target cells) of treated patients [42].

Calibration curves of TKI were performed from 1 nM to 1 µM and TKI LC-MS peak area versus concentrations showed linear regression fitting. Most pure standards of TKI metabolites were not commercially available and proper calibration curves could not be done, preventing the absolute quantification and determinations of metabolite formation rate in universal units. Therefore, the velocities of metabolite production were calculated in arbitrary units (*arb*) as LC-MS peak area/time. Using GraphPad Prism software version 4, a nonlinear regression analysis was performed for the determination of the kinetic constants: affinity, Km in µM, maximum velocity Vmax in *arb* and intrinsic clearance, IC ( = Vmax/Km) in *arb*. Km and Vmax constant determinations for all 4 CYP were performed the same day and analyzed at the same time.

### Cell line

The human hepatoma cell line (HepG2) was grown in Dulbecco's Modified Eagle Medium containing 10% fetal bovine serum (FBS) and supplemented with non essential amino acid, penicillin (200 UI/mL) and streptomycin (50 µg/mL).

### Recombinant adenovirus (Ad)

CYP1A1, CYP1B1, CYP2J2 and CYP 3A4 cDNA provided by our laboratory [43] were sent to the University Hospital of Nantes supported by the *Association Française contre la Myopathie* and cloned into serotype five adenovirus [44]. A replication defective E1 and E3 region deleted adenovirus encoding lacZ (control), CYP1A1, CYP1B1, CYP2J2 and CYP 3A4 were constructed and purified, the titer was determined with a spectrophotometer.

### Adenoviral infection and biotransformation assays

HepG2 cells were seeded in six-well plates at 400,000 cells/well. Twenty four hours later, the cells were incubated for 4 hours with Ad at a concentration of 300 multiplicity of infection (MOI), in cell culture medium containing 2% FBS. After 4 hours, the Ad-containing medium was removed and replaced with normal HepG2 medium. Ad infected cells were treated with 1 µM TKI in media for 72 hours, control cells were teated with DMSO (0.1%). Cultured medium from the cells incubated with or without TKI, was collected at 0, 6, 24, 48 and 72 hours for analysis of disappearance of TKI and appearance of TKI metabolite by LC-MS assays.

### Sample preparation

Incubations or culture media samples were extracted by protein precipitation with 2 volumes of MeCN and centrifugated (16,000 g for 10 min). Protein pellets were discarded. Then, supernatants were 2× diluted with 10 mM ammonium formate in water before the injection of 10 µL onto the LC-MS system. Using this extraction procedure, the extraction yields of TKI were ≥90% [45]. Calibration curves of TKI were performed to ensure curve linearity and procedure quality.

### LC-MS system and conditions

The LC-MS system consisted in a Rheos 2200 pump (Flux Instruments, Switzerland), a PAL autosampler (CTC Analytics, Switzerland), an electrospray ionization source in positive mode (ESI^+^) and a triple quadrupole mass spectrometer, TSQ Quantum discovery, from Thermo (USA) and was monitored by Xcalibur software (Thermo, USA). ESI sheath and auxiliary gas (nitrogen) flow-rates were set at 40 psi and 10 arbitrary units, respectively. The capillary voltage and heated capillary temperature were set at +4 kV and 300°C, respectively. Tube lens voltages ranged from 120 to 180 V.

A BEH C18 analytical column (2.1×50 mm; id×length; particle size 3.5 µm; Waters, USA) was used. Mobile phase consisted of A: 0.1% formic acid and B: MeCN+0.1% formic acid. Depending on the experiment type, different gradient slopes were used. Typically, gradient was as follows: 0 min: 5% of B, 5 min: 80% of B, 7 min: 95% of B followed by 2 min of cleaning (98% B) and 2 min of equilibration (5% of B). Flow rate was 0.35 mL/min. A typical LC-MS chromatogram is depicted in **Figure S2**. For the determination of imatinib metabolites, a HILIC analytical column (2.1 mm×50 mm length; particle size 3.5 µm; Waters, USA) was used. Mobile phase consisted of A: 10 mM ammonium formate+0.1% formic acid and B: MeCN+0.1% formic acid. Gradient was as follows: 0 min: 90% of B hold for 0.5 min, 6 min: 50% of B followed by 3 min of cleaning (50% B) and 3.5 min of equilibration (90% B). Flow rate was 0.35 mL/min. Injection volume was 10 µL on both columns.

Identification of TKI metabolites in our LC-MS analyses has been done following methodologies described previously and considering previously published data [15], [46], [47]. Briefly, extracts of *in vitro* incubations were injected in our LC-MS system. Product ion scan and data dependant scan acquisitions were performed. The presence of ≥2 similar ions between metabolite and parent drug in MS^2^ spectra as well as the increase of metabolite amounts over time confirmed the identification. TKI and their metabolites were detected by selective reaction monitoring (SRM). Ion transitions and collision energies are given in **Figure S2**.

### Human samples and mRNA expression

Human samples were provided by the Biobank of Lausanne (CHUV hospital). The research protocol was approved by both a scientific and a local ethics committee (see below **). All patients have consented to participate. Protocole 195/10: Title: “Expression et activité des enzymes du métabolisme des médicaments dans les tissus tumoraux”. The participants provided their written and informed consent to participate in this study. Written participant consents have been collected for every patient. The ethics committees/IRBs approved this consent procedure. (**) *Commission Cantonale d'éthique de la recherche sur l'être humain*. Route du Bugnon. CH-1011 Lausanne (link: www.unil.ch/fbm/page36053.html).

Patients' tumors were removed according to medical decisions and surgical practice. When possible, a part of the resected tissue was cut into tumor tissues and healthy tissues surrounding the tumor (≈2 g each), and rapidly frozen intact into liquid nitrogen and stored at −80°C. All tissues were evaluated by a pathologist who observed tissue sections with a microscope. Only the tissues containing ≥∼85% of cancer cells were kept for this study. Tissues containing ≥∼90% healthy cells were considered as the healthy counterpart. Fibrous, fat or >40% necrotic tissues were discarded. Fourteen renal cell carcinoma (RCC) and six hepatocellular carcinoma (HCC) tumors and their surrounding healthy tissues were used for the determination of CYP mRNA expression. Most patients did not receive anticancer drugs prior surgery (See **Table S1**).

### Real-time reverse transcriptase-PCR

Total RNA was extracted from frozen tissues using a modified guanidine-thiocyanate method. After determination of the quantity of isolated RNA using a NanoDrop ND-1000 spectrophotometer (NanoDrop Technologies), cDNA was prepared from 500 ng of total RNA in 25 µl of reaction volume, using high capacity cDNA archive kit (Applied Biosystems).

### Quantitative real-time PCR

Total mRNA expression is given as N-fold differences in CYP gene expression (Ncyp) relative to the TBP gene. The Ncyp values of the samples were subsequently normalized to a basal mRNA level, the smallest amount of CYP gene mRNA quantifiable and scored as the smallest Ncyp value. The methodology was previously described [5].

### Study Highlights

- The study investigates the capability of the 3 *extrahepatic* CYP1A1, 1B1 and 2J2 to biotransform 5 tyrosine kinase inhibitors (TKI) and measures their RNA expression in resected tumor biopsies and their healthy tissue counterparts.
- The results show that CYP2J2 can biotransform most TKIs tested with metabolic efficiency comparable to that of CYP3A4. CYP2J2 was highly expressed in 30% of renal cancer carcinoma biopsies whereas poorly expressed in the healthy tissue counterparts and liver.
- Our results suggest that a specific inhibition of CYP2J2 activity in patients could significantly increase *intratumoral* TKI disposition without modifying plasma levels. CYP2J2 inhibitors that have already been identified among approved drugs, could be co-administered to possibly improve clinical outcome.

## Supporting Information

Figure S1 **A) LC-MS chromatogram of nilotinib (top) and 2 metabolites (below), N-oxide-nilotinib (NO-nilotinib) and hydroxy-nilotinib (OH-nilotinib).** MS^2^ product scan of nilotinib (insert in the above chromatogram) show typical ions (highlighted with a (✶)) that are found in MS^2^ product scan of the 2 metabolites. B) Velocity of metabolite production (V in arbitrary unit, (arb)) of NO- and OH-nilotinib against nilotinib concentration (in µM) in microsome incubation with cDNA expressed CYP2J2 and 3A4 isozymes. Fitting of the curve by dedicated software following Michaelis-Menten equation allowed the determination of the affinity constants (Km in (µM)), the maximum velocities (Vmax in (arb)) and the intrinsic clearance (IC in (arb)).
(TIF)

Figure S2 **Left. LC-MS chromatogram of TKI.** Right. TKI and metabolites determined with ion transitions. *Abbreviations*: D; deuterium; IS, internal standards, NO-, N-oxide; OH-; hydroxy-; RT, retention time; TKI, tyrosine kinase inhibitors.
(TIF)

Table S1 **Number of transcripts of CYP1A1, 1B1, 2J2 and 3A4 mRNA in renal cell carcinoma (RCC, top) and hepatocellular carcinoma (HCC, bottom) tumor tissues and healthy tissue counterparts withdrawn from cancer patients.** Using the similar protocol than in Bièche et al. [5], these relative values can be compared.
(TIF)
