# Supplementary figures and images for: Important Role of CYP2J2 in Protein Kinase Inhibitor Degradation: A Possible Role in Intratumor Drug Disposition and Resistance
Source: PLoS One. 2014 May 12;9(5):e95532. doi: 10.1371/journal.pone.0095532 (PMC4018390; doi:10.1371/journal.pone.0095532)

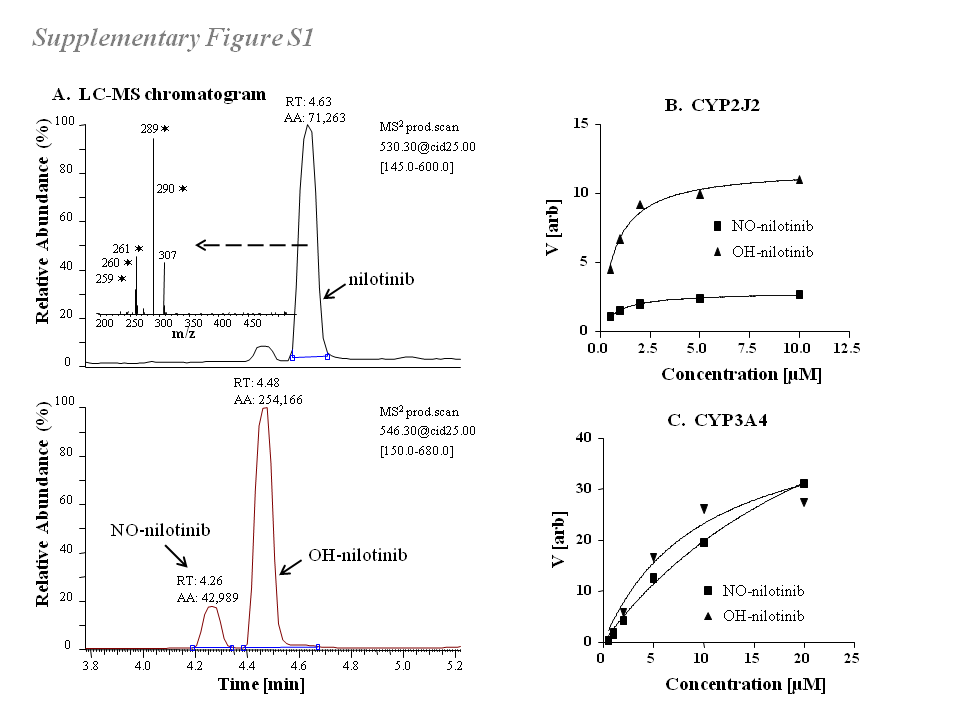

Supplement: Figure S1 — A) LC-MS chromatogram of nilotinib (top) and 2 metabolites (below), N-oxide-nilotinib (NO-nilotinib) and hydroxy-nilotinib (OH-nilotinib). MS2 product scan of nilotinib (insert in the above chromatogram) show typical ions (highlighted with a (✶)) that are found in MS2 product scan of the 2 metabolites. B) Velocity of metabolite production (V in arbitrary unit, (arb)) of NO- and OH-nilotinib against nilotinib concentration (in µM) in microsome incubation with cDNA expressed CYP2J2 and 3A4 isozymes. Fitting of the curve by dedicated software following Michaelis-Menten equation allowed the determination of the affinity constants (Km in (µM)), the maximum velocities (Vmax in (arb)) and the intrinsic clearance (IC in (arb)). (TIF) [file pone.0095532.s001.tif]

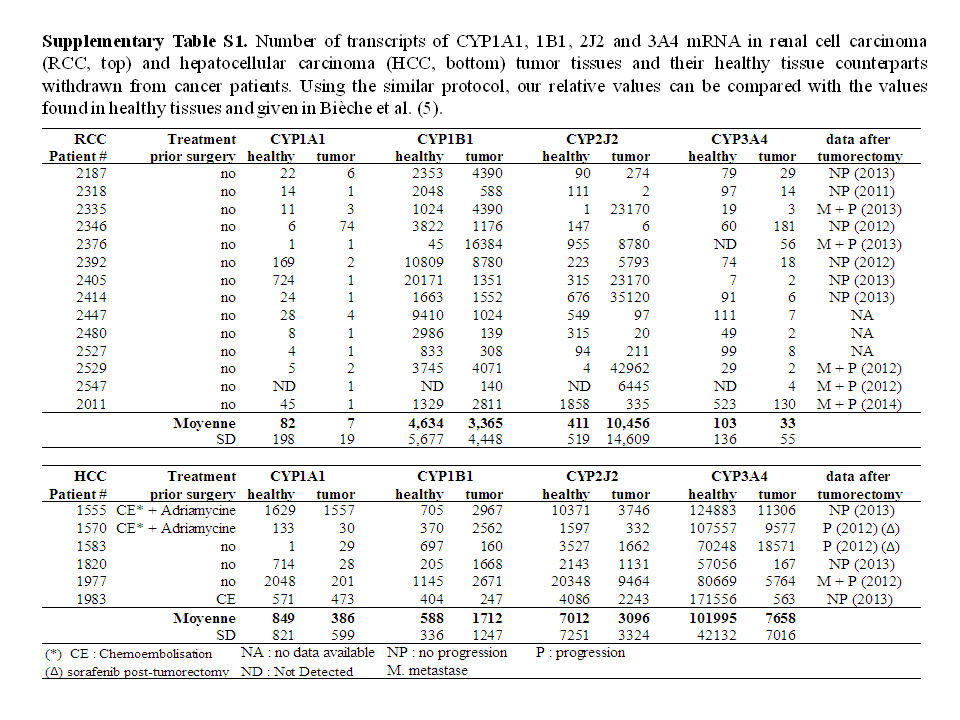

Supplement: Table S1 — Number of transcripts of CYP1A1, 1B1, 2J2 and 3A4 mRNA in renal cell carcinoma (RCC, top) and hepatocellular carcinoma (HCC, bottom) tumor tissues and healthy tissue counterparts withdrawn from cancer patients. Using the similar protocol than in Bièche et al. [5], these relative values can be compared. (TIF) [file pone.0095532.s003.tif]
